# Identification and Validation Prognostic Impact of MiRNA-30a-5p in Lung Adenocarcinoma

**DOI:** 10.3389/fonc.2022.831997

**Published:** 2022-01-20

**Authors:** Xiulin Jiang, Yixiao Yuan, Lin Tang, Juan Wang, Dahang Zhang, William C. Cho, Lincan Duan

**Affiliations:** ^1^ The Department of Thoracic Surgery, The Third Affiliated Hospital of Kunming Medical University, Kunming, China; ^2^ Key Laboratory of Animal Models and Human Disease Mechanisms of Chinese Academy of Sciences, Kunming Institute of Zoology, Kunming, China; ^3^ Department of Clinical Oncology, Queen Elizabeth Hospital, Hong Kong, Hong Kong SAR, China

**Keywords:** miRNA-30a-5p, NSCLC, prognosis biomarker, cell proliferation, cell migration

## Abstract

MiRNA-30a-5p is a microRNA found to be decreased in various human cancers, including lung adenocarcinoma (LUAD). However, the molecular mechanisms of miRNA-30a-5p involve in the progression of LUAD remains unclear. In this study, we found that miRNA-30a-5p expression was significantly decreased in LUAD cells lines, LUAD tissues, and peripheral blood serum. Besides, LUAD patients with decreased miRNA-30a-5p expression exhibit worse clinical outcomes compared to the patients with higher miRNA-30a-5p expression, decreased expression of miRNA-30a-5p was associated with advanced clinical outcomes. Receiver operating characteristic (ROC) curve analysis of miRNA-30a-5p showed an area under the curve (AUC) value of 0.902, indicating its prognostic value in LUAD. Moreover, immune infiltration and gene set enrichment analysis (GSEA) enrichment analyze demonstrated that miRNA-30a-5p expression was associated with immune cell infiltrated in LUAD. Finally, we found that miRNA-30a-5p inhibits cell proliferation, migration, and self-renewal abilities of LUAD *in vitro*. In summary, this is the first report that miRNA-30a-5p correlated with progression and immune infiltration, which shed some lights on potential prognostic and therapeutic biomarker for LUAD.

## Introduction

Lung cancer is a malignancy that originates in the bronchial mucosa or glands of the lungs. As cancer cells grow and spread, they severely damage the patient’s respiratory system and compromise oxygen exchange (1). As the tumor with the highest mortality, lung cancer mainly includes small cell lung cancer and non-small cell lung cancer (NSCLC), the NSCLC is comprised of adenocarcinoma (AC), squamous cell carcinoma (SCC), and large-cell carcinoma (LCC) (1). Compared with SCLC, LUAD usually grows and spreads more slowly. For patients diagnosed with early stage LUAD, tumors can usually be resected surgically (2). When the tumor has metastasized locally, it is treated by the simultaneous use of radiotherapy and chemotherapy (1). For the last decade, limited by the treatment options, many endeavors targeting various signaling pathways and putative driver mutations, as well as angiogenesis mechanisms, have been carried out to improve the clinical outcome. However, the overall survival time of lung cancer remains measured in months. Therefore, it is urgent that develop effective therapeutic strategies to improve the survival time of lung patients.

As one of noncoding RNA, MicroRNAs (miRNAs) refers to a class of short endogenously noncoding RNAs that negatively regulate mRNA expression by binding the complementary sequences in the target genes 3′-untranslated region (UTR) (3). Emerging evidence has demonstrated that miRNAs play crucial roles in cancer development and progression (2). The miR-30 family is composed of miR-30a, miR-30b, miR-30c, miR-30d, and miR-30e, which play different roles in regulating cancer progression (3). MiR-30a-5p is a member of the miR-30 family and has been reported to be located in the genome-vulnerable region of lung cancer (4). Mounting evidence indicated that miRNA participates in the cancer hallmarks *via* activation of diverse oncogenes and growth enhancers (4). For example, it has been shown that miRNA−30a−3p inhibits the expression of IGF−1R and result in reduced cell migration ability of esophageal carcinoma (5). In hepatocellular carcinoma, the study has reported that miR-30a markedly decreased in hepatocellular carcinoma (HCC) tissues and cell lines, overexpression of miR-30a restrain the lung cancer cell proliferation and migration, a further study has shown that miR-30a by down-regulated the expression of Atg5 and lead to inhibits hepatocellular carcinoma (HCC) metastasis (6). However, the potential roles of miR-30a-5p in regulation the lung cancer stem cell maintenance and tumor microenvironment were unclear.

In this study, we compared the expression of miR-30a-5p between LUAD tissues and normal samples, and investigated the correlation between miR-30a-5p expression and clinical parameters of LUAD. In addition, we explored the prognostic value and clinical significance of miR-30a-5p in LUAD. Meanwhile, the correlation between miR-30a-5p expression and immune infiltration was analyzed using ssGSEA (single-sample Gene Set Enrichment Analysis) to explore the potential mechanisms involved in miR-30a-5p modulation in the carcinogenesis of LUAD. Finally, the biological role of miR-30a-5p was identified in LUAD. In summary, we demonstrated that the potential role of miR-30a-5p in regulating tumor progression and its potential application in the diagnosis and prognostic evaluation in LUAD. qRT-PCR, growth curve, colony formation, tumor sphere, transwell and Wound healing assay used to determine the function of miR-30a-5p in LUAD progression. Our findings underline the vital role of miR-30a-5p in LUAD prognosis. We found that forced miR-30a-5p significantly inhibited the LUAD cell proliferation, migration, and self-renewal abilities of LUAD. Also, we provide an underlying mechanism of miR-30a-5p expression in potentially regulating the infiltration of immune cells partly affecting the prognosis of LUAD.

## Materials and Methods

### Data Collection

TCGA-LUAD cohort data and corresponding clinical information of 535 LUAD patients were downloaded from the TCGA website (https://portal.gdc.cancer.gov/repository). The gene expression profiles were normalized using the scale method provided in the “limma” R package. Data analysis was performed with the R (version 3.6.3) and ggplot2 [3.3.3] packages. The expression data were normalized to transcripts per kilobase million (TPM) values before further analysis.

### Nomogram Construction and Evaluation

Nomogram Construction was performed as previously described (5). Based on the multivariate Cox analysis results, we established a nomogram to predict the prognosis of lung adenocarcinoma patients. According to the prognosis model, we calculated each patient’s risk score as the total score of each parameter, which could predict the prognosis of lung adenocarcinoma patients. The accuracy estimation of nomogram prediction was obtained from a calibration plot. It was found that the bias-corrected line in the calibration plot was close to the ideal curve (Keynesian cross), indicating a strong consistency between predicted values and observed values. The nomogram discrimination was determined using a concordance index (C-index), and 1,000 resamples were used in calculation by bootstrap approach. In this study, all statistical tests were two-tailed, with a statistical significance level of 0.05. The discrimination ability of miRNA-30a-5p in LAUD was evaluated through receiver operating characteristic (ROC) analysis using the pROC package (6).

### Gene Set Enrichment and Immune Infiltration Analysis

The target genes of miRNA-30a-5p were predicted by starbase (https://starbase.sysu.edu.cn/) (7). The GO and Kyoto Encyclopedia of Genes and Genomes (KEGG) pathway enrichment analyses were performed for the target gene of miRNA-30a-5p using the clusterProfiler package (8).We also utilized the cluster Profiler package and GSEA software to analyze the potential signaling pathway and molecular function in LUAD (8, 9). We used a GSVA R package to examine the LUAD immune infiltration of 24 tumor-infiltrating immune cells in tumor samples through ssGSEA (10, 11). The correlation between miRNA-30a-5p and immune infiltration levels was analyzed by the Spearman correlation, and these immune cells with the different expression groups of miRNA-30a-5p were analyzed by the Wilcoxon rank-sum test.

### StarBase V2.0 Analysis

StarBase v2.0 (http://starbase.sysu.edu.cn/) an systematically identify the RNA-RNA and protein-RNA interaction networks from 108 CLIP-Seq (PAR-CLIP, HITS-CLIP, iCLIP, CLASH) data sets generated by 37 independent studies. By combining 13 functional genomic annotations, we developed miRFunction and ceRNAFunction web servers to predict the function of miRNAs and other ncRNAs from the miRNA-mediated regulatory networks. In this study, we used starbase to predicte the target gene of miRNA-30a-5p.

### Kaplan-Meier Plotter Database Analysis

We used KM Plotter (http://kmplot.com), an online database that contains gene expression data and survival information of 3452 clinical lung cancer patients, to analyze the prognostic value of miRNA-30a-5p in pan-cancer cancer. The patient samples were separated into two groups by median expression (high expression and low expression) and diverse immune cell (increased and decreased group) to analyze the overall survival (OS), progression-free survival (PFS) and postprogression survival (PPS) with hazard ratios (HRs) with 95% confidence intervals (95% CIs) and log-rank p-values.

### Cell Culture and Micro-RNA Transfection

BEAS-2B cell line was purchased from Cell Bank of Kunming Institute of Zoology, and cultured in BEGM media (Lonza, CC-3170). Lung cancer cell lines, including A549, H1299, and SPC-A1, were purchased from Cobioer, China with STR document, and were cultured in RPMI-1640 medium (Corning) supplemented with 10% fetal bovine serum (FBS) and 1% penicillin/streptomycin. The NC control and miRNA-30a-5p mimics were purchased from RiboBio (China). Cells were transfected with indicated miRNA mimics or control NC using Lipofectamine 3000 (Invitrogen), and then collected for various experiments.

### Cell Proliferaion Assay

For the colony formation assay, 500 cells were seeded on soft agar in 6-well plates, and colonies were counted 3 weeks after seeding. The resulting colonies were then washed twice with PBS and fixed with 4% formaldehyde for 30 min and stained for 12 hours with 0.01% crystal violet. The number of colonies was then counted. For the BrdU incorporation assay, 20 min before fixation, indicated cells were pre-treated with 10 588 μM BrdU (Abcam, ab142567, 1:100), and fixed with 4% PFA followed by BrdU primary antibody staining (CST, 5292s, 1:1000), then further stained by secondary antibody (Abclonal, 61303, 1:500). DAPI was used to stain the cell nuclei. More than five fields/samples randomly selected were imaged by a Nikon Ti fluorescence microscope and quantified.

### Cell Migration Assay

Cell migration assay was performed as previously described (12). Briefly, indicated cells were seeded into 6-well plates (1×10^6^/cell) and incubated for one day, and then a straight line was scraped with pipette tips. Detached cells were removed. Photographs were taken at the indicated time, and the relative traveled distance was measured. For the trans-well migration assay, 3×10^4^ cells/well in 100μL serum-free medium were plated in a 24-well plate chamber insert, and the lower chamber was filled with 10% FBS. After incubation for 24 h, cells were fixed with 4% PFA, washed, and then stained with 0.5% crystal violet for further pictures captured.

### Tumor Sphere Formation

Cells 3×10^4^ well were plated in ultralow-attachment 6 well plates (Corning; 3471) and grown in serum-free DMEM/F12, supplemented with B27, 20 ng/mL EGF and 20 ng/mL bFGF, and 4 μg/mL heparin. The spheres were cultured for 14 days, and then pictured and counted.

### Real-Time RT-PCR Assay

The qRT-PCR assay was performed as documented (13). For the qRT-PCR assay, indicated total RNAs were extracted from cells using RNAiso Plus (Takara, 108-95-2), following which they were reverse transcribed using a PrimeScript RT reagent Kit (Takara Bio, RR047A). cDNA was subjected to RT-qPCR analysis using FastStart Universal SYBR Green Master Mix (Roche, 04194194001). All reactions were performed in triplicate using an Applied Biosystems 7500 machine. The primer sequences are list follows MiRNA-30a-5p-F: GGGCCTGTAAACATCCTCG, miRNA-30a-5p-R: GAATACCTCGGACCCTGC, U6-F: GGTCGGGCAGGAAAGAGGGC, U6-R: GCTAATCTTCTCTGTATCGTTCC, SOX2-F:CACAGATGCAACCGATGCA, SOX2-R:GGTGCCCTGCTGCGAGTA, CD44-F: CTGCCGCTTTGCAGGTGTA, CD44-R: CATTGTGGGCAAGGTGCTATT, NANOG-F: TTTGTGGGCCTGAAGAAAACT, NANOG-R: AGGGCTGTCCTGAATAAGCAG, OCT4-F: CTGGGTTGATCCTCGGACCT and OCT4-R: CCATCGGAGTTGCTCTCCA. The expression quantification was obtained with the 2−ΔΔCt method.

### Statistical Analysis

Correlation analysis was performed using the Pearson correlation test. Kaplan-Meier survival curves were plotted to exhibit the overall survival for LUAD patients. We employed The Wilcoxon rank-sum

Test and Chi-square test examine the correlation between miRNA-30a-5p and clinical features. Univariate and multivariate Cox regression analyses were used to examine the independent prognostic significance of each variable enrolled in this finding. For the function of miRNA-30a-5p in LUAD, GraphPad Prism 7.00 was utilized for statistical analyses. The significance of the data between the two experimental groups was determined by Student’s t-test, and multiple group comparisons were analyzed by one-way ANOVA. P < 0.05 (*), P < 0.01 (**) and P < 0.001 (***), were considered significant.

## Results

### MiRNA-30a-5p Is Decreased in Pan-Cancer

**Figure 1:**
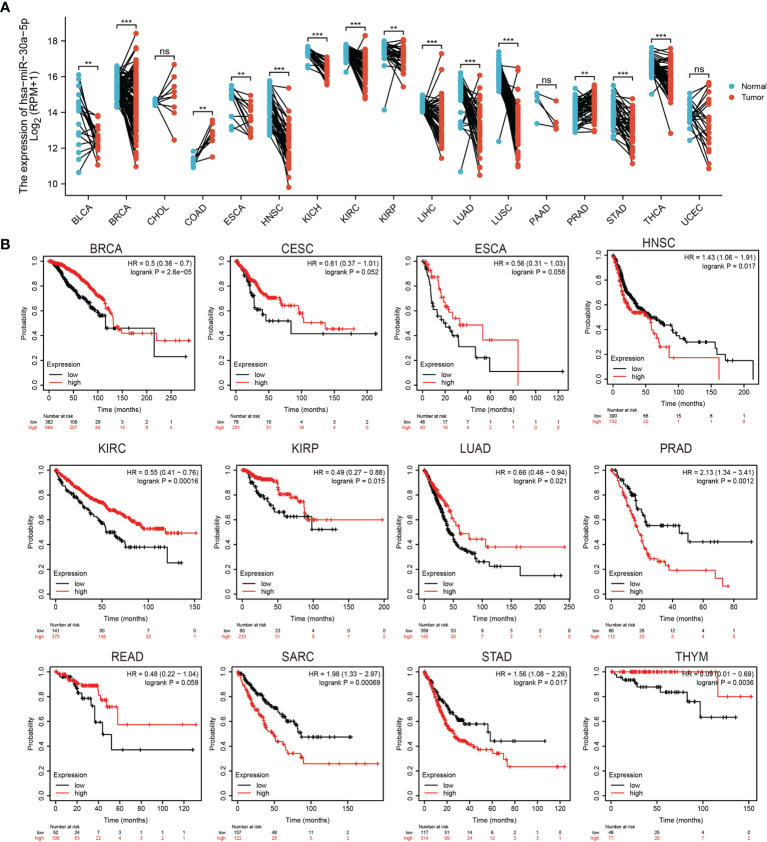
Expression analysis for miRNA-30a-5p in human cancers. **(A)** The expression of miRNA-30a-5p in pan-cancer. **(B)** The overall survival of miRNA-30a-5p in pan-cancer analysed by the Kaplan-Meier Plotter database. **P < 0.01, ***P < 0.001, NS: p >0.05.

To examine the expression of miRNA-30a-5p in diverse human cancer, we utilized starbase to examine its expression pattern, the results demonstrated that miRNA-30a-5p decreased in bladder urothelial carcinoma (BLCA), breast invasive carcinoma (BRCA), esophageal carcinoma(ESCA), head and neck squamous cell carcinoma (HNSC), kidney chromophobe (KICH), kidney renal clear cell carcinoma (KIRC), kidney renal papillary cell carcinoma (KIRP), liver hepatocellular carcinoma (LIHC), lung adenocarcinoma (LUAD), lung squamous cell carcinoma (LUSC), stomach adenocarcinoma and (STAD), thyroid carcinoma (THCA) (**Figure 1A**). On the contrary, the high expression of miRNA-30a-5p was observed in colon adenocarcinoma (COAD) and prostate adenocarcinoma (PRAD). Taken together, these results demonstrate that miRNA-30a-5p was down-regulated in most human cancer.

To uncover that the prognostic value of miRNA-30a-5p in pan-cancer, we perform the OS analysis in human cancers, the result showed that miRNA-30a-5p high expression correlated with better overall survival in BRCA, CESC, ESCA, KIRC, KIRP, LUAD, READ, and THYM, as well as associated with the shorter disease-free survival in HNSC, PRAD, SARC and STAD (**Figure 1B**).

### Analysis of the Diagnosis Value of MiRNA-30a-5p in Diverse Human Cancer

**Figure 2:**
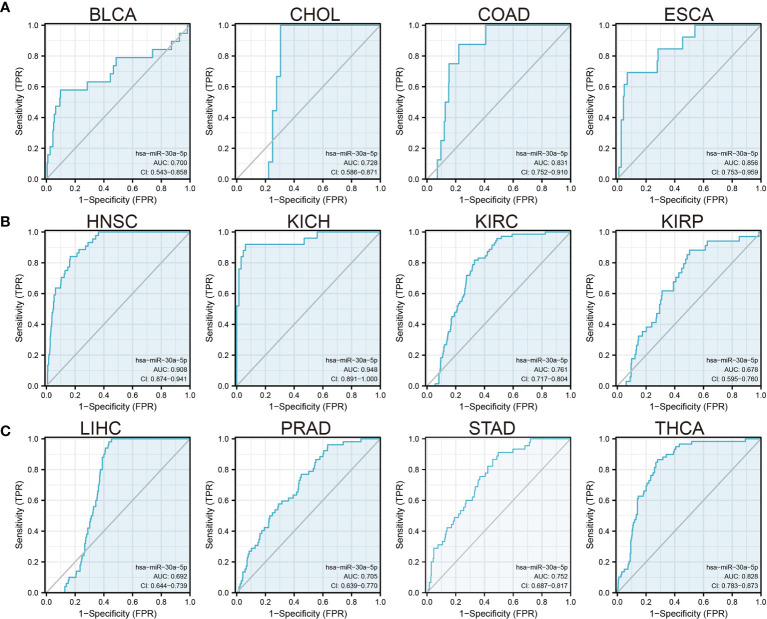
Analysis of the ROC curve for miRNA-30a-5p in human cancers. **(A–C)** ROC curves of miRNA-30a-5p for predicting overall survival in diverse cancer patients.

We previously showed that miRNA-30a-5p was the low expression and correlated with prognosis in several cancers, we, therefore, examined whether miRNA-30a-5p act as a detection index for the diagnosis of diverse cancer, ROC curve analysis of miRNA-30a-5p showed an area under the curve (AUC) value of 0.700 in BLCA, the AUC value of 0.728 in CHOL, the AUC value of 0.831 in COAD, the AUC value of 0.856 in ESCA, the AUC value of 0.908 in HNSC, the AUC value of 0.948 in KICH, the AUC value of 0.761 in KIRC, the AUC value of 0.678in KIRP, the AUC value of 0.692 in LIHC, the AUC value of 0.705 in PRAD, the AUC value of 0.752 in STAD and the AUC value of 0.828 in THCA (**Figures 2A–C**). These results confirmed that miRNA-30a-5p has a higher diagnostic ability for the diagnosis of various cancer.

### Analysis of the Function of Target Genes of MiRNA-30a-5p

**Figure 3:**
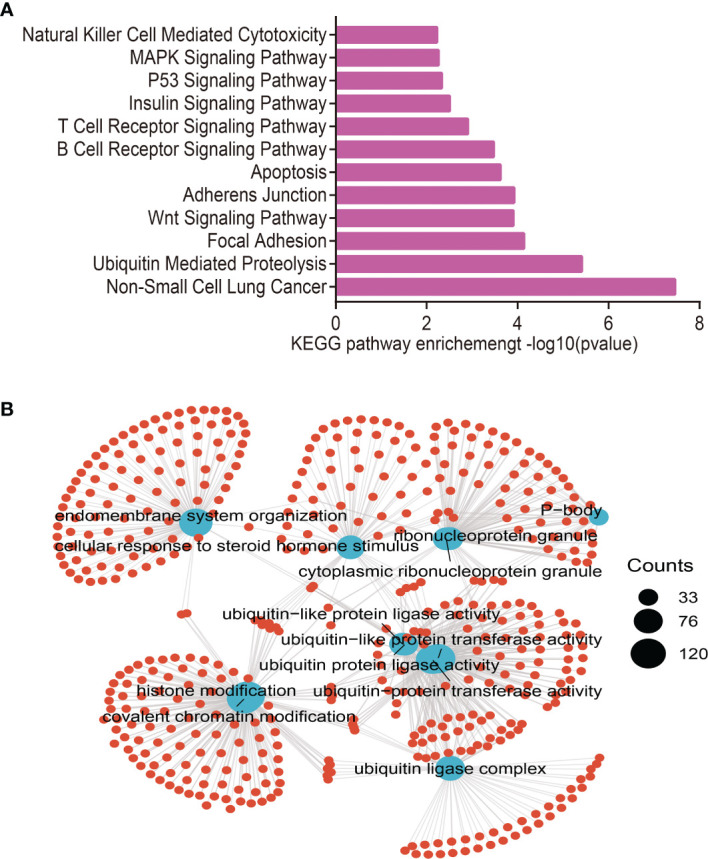
Analysis of the biological function for miRNA-30a-5p downstream target genes in human cancers. **(A)** The KEGG signaling pathway of miRNA-30a-5p downstream target genes in pan-cancer analysis by the starbase database. **(B)** The biological process of miRNA-30a-5p downstream target genes in pan-cancer analysis by the starbase.

Considering the miRNA-30a-5p was markedly related to the prognosis, tumor stage, and lymph node metastasis, we next explored the functions of a miRNA-30a-5p target gene in cancer, we utilized the starbase and targets can databases to obtain the target gene of miRNA-30a-5p, and using this gene to perform the gene ontology (GO) and the Kyoto Encyclopedia of Genes and Genomes (KEGG) enrichment analysis. The analysis revealed that the target gene of miRNA-30a-5p is mainly involved in signaling pathway including the Non-Small Cell Lung Cancer, Ubiquitin Mediated Proteolysis, Focal Adhesion, Wnt Signaling Pathway, Adherens Junction, apoptosis, B Cell Receptor Signaling Pathway, T Cell Receptor Signaling Pathway, Insulin Signaling Pathway, P53 Signaling Pathway, MAPK Signaling Pathway and Natural Killer Cell-Mediated Cytotoxicity (**Figure 3A**). The miRNA-30a-5p target gene is mainly involved in the biology process including the endomembrane system organization, cellular response to steroid hormone stimulus, cytoplasmic ribonucleoprotein granule, ubiquitin-like protein ligase activity, ubiquitin ligase complex, histone modification, and covalent chromatin modification (**Figure 3B**). These findings suggested that the target gene of miRNA-30a-5p plays a pivotal role in immune responses and cancer progression.

### Identification of MiRNA-30a-5p Target Gene-Associated Signaling Pathways Using GSEA

**Figure 4:**
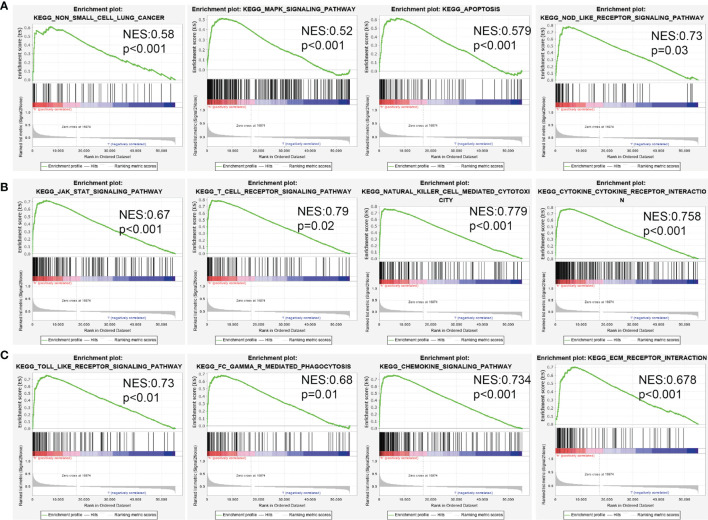
Analysis of the signaling pathway for miRNA-30a-5p target genes in human cancers. **(A–C)** The potential signaling pathway of miRNA-30a-5p target gene analysed using GSEA software.

To uncover the potential mechanism related to the miRNA-30a-5p target gene expression, we further explored the most significant enrichment signaling pathways with miRNA-30a-5p target gene expression by utilizing GSEA software (9), the results demonstrated that the miRNA-30a-5p target gene expression mainly involved in Non-small cell lung cancer, apoptosis, chemokine signaling pathway, cytokine-cytokine receptor interaction, ECM receptor interaction, INFγ mediated phagocytosis, JAK-STAT signaling pathway, MAPK signaling pathway, Natural killer cell-mediated cytotoxicity, NOD-like receptor signaling pathway, T cell receptor signaling pathway, and Toll-like receptor signaling pathway (**Figures 4A–C**).

### Analysis of the Correlation Between MiRNA-30a-5p Expression and Clinicopathologic Characteristic

**Figure 5:**
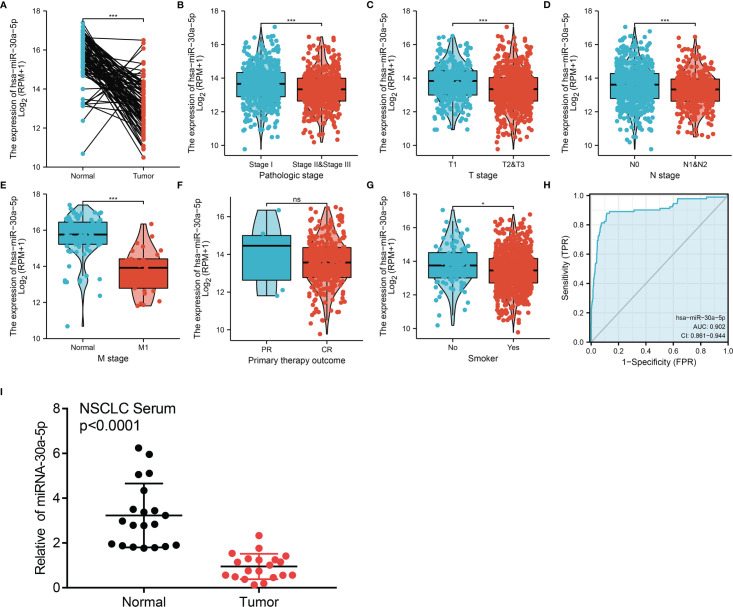
miRNA-30a-5p was down-regulated in LUAD. **(A)** MiRNA-30a-5p was down-regulated in lung cancer based on TCGA-LAUD dataset. **(B–G)** Correlation between miRNA-30a-5p and clinicpathologic features, including the pathology stage, TNM stage, smoker, and therapy outcome in LUAD. **(H)** ROC curve analysis of miRNA-30a-5p showed an AUC value of 0.902 in LUAD. **(I)** miRNA-30a-5p was down-regulated in peripheral blood serum of LUAD analysed using qRT-PCR assay. *P < 0.05, ***P < 0.001. CR, complete response; PR, partial response; SD, stable disease; PD, progressive disease. NS: p >0.05.

In the TCGA LUAD dataset, miRNA-30a-5p is down-regulated in LUAD (**Figure 5A**). Correlation analysis was used to identify the clinic-pathologic characteristic and miRNA-30a-5p expression level. As shown in **Table 1** and **Figures 5B–G**, low expression of miRNA-30a-5p was significantly correlated with the TNM stage, smoking, and age (p < 0.05). Using univariate analysis, as a categorical dependent variable, the expression of miRNA-30a-5p was correlated with TNM stage, pathologic stage, Gender, Age, and Smoker (**Table 2**). The ROC curve analysis of miRNA-30a-5p showed an AUC value of 0.902, indicating its prognostic value in LUAD (**Figure 5H**), the down-regulation of miRNA-30a-5p was verified in paired peripheral blood serum (n=20), using real-time quantitative-PCR compared with reciprocal controls (**Figure 5I**). As shown in **Table 3**, LUAD patients having complete clinical data were included in further Cox regression analysis. In the Cox univariate regression analysis, lower expression of miRNA-30a-5p, TN stage, pathologic stage, residual tumor, and primary therapy outcome were correlated with overall survival in LUAD patients.

**Table 1:** Analysis of the correlation between miR-30a-5p expression and clinic-pathologic features in the TCGA LUAD dataset.

Characteristic	Low expression of hsa-miR-30a-5p	High expression of hsa-miR-30a-5p	p
n	499	500	
T stage, n (%)			< 0.001
T1	108 (10.8%)	171 (17.2%)	
T2	303 (30.4%)	255 (25.6%)	
T3	70 (7%)	48 (4.8%)	
T4	17 (1.7%)	24 (2.4%)	
N stage, n (%)			0.008
N0	296 (30.2%)	341 (34.8%)	
N1	128 (13.1%)	94 (9.6%)	
N2	66 (6.7%)	48 (4.9%)	
N3	3 (0.3%)	3 (0.3%)	
M stage, n (%)			0.493
M0	368 (47.8%)	373 (48.4%)	
M1	12 (1.6%)	17 (2.2%)	
Age, meidan (IQR)	66 (59, 72)	68 (62, 74)	< 0.001

**Table 2:** miR-30a-5p expression correlated with clinical pathological characteristics (logistic regression).

Characteristics	Total (N)	Odds Ratio (OR)	P value
T stage (T2&T3&T4 vs. T1)	996	0.530 (0.399-0.701)	<0.001
N stage (N1&N2&N3 vs. N0)	979	0.639 (0.490-0.832)	<0.001
M stage (M1 vs. M0)	770	1.398 (0.663-3.038)	0.383
Primary therapy outcome (PD&PR vs. SD&CR)	782	0.683 (0.450-1.027)	0.069
Pathologic stage (Stage III&Stage IV vs. Stage I&Stage II)	988	0.746 (0.543-1.022)	0.049
Race (White vs. Asian)	744	0.718 (0.258-1.889)	0.505
Gender (Male vs. Female)	999	0.768 (0.596-0.990)	0.042
Age (>65 vs. <=65)	972	1.448 (1.123-1.871)	0.004
Residual tumor (R1&R2 vs. R0)	752	0.633 (0.295-1.309)	0.225
Smoker (Yes vs. No)	974	0.652 (0.418-1.005)	0.050

**Table 3:** Univariate regression and multivariate survival model of prognostic covariates in patients with LUAD.

Characteristics	Total(N)	Univariate analysis		Multivariate analysis	
		Hazard ratio (95% CI)	P value	Hazard ratio (95% CI)	P value
T stage	981				
T1	279				
T2	546	1.372 (1.064-1.769)	0.015	0.945 (0.640-1.394)	0.775
T3&T4	156	2.438 (1.791-3.318)	<0.001	2.061 (1.184-3.585)	0.011
N stage	964				
N0	629				
N1	218	1.558 (1.232-1.970)	<0.001	1.132 (0.778-1.647)	0.516
N2&N3	117	2.115 (1.593-2.808)	<0.001	2.159 (1.036-4.497)	0.040
M stage	757				
M0	728				
M1	29	2.461 (1.560-3.883)	<0.001	2.135 (0.927-4.918)	0.075
hsa-miR-30a-5p	984	0.917 (0.835-1.008)	0.043	0.967 (0.836-1.118)	0.647
Pathologic stage	973				
Stage I&Stage II	783				
Stage III&Stage IV	190	2.103 (1.683-2.628)	<0.001	0.758 (0.375-1.531)	0.440
Primary therapy outcome	771				
SD&CR	664				
PD&PR	107	4.010 (3.049-5.273)	<0.001	5.218 (3.420-7.961)	<0.001
Residual tumor	739				
R0	709				
R2&R1	30	3.016 (1.863-4.882)	<0.001	1.654 (0.759-3.602)	0.205
Smoker	959				
No	89				
Yes	870	0.920 (0.640-1.324)	0.654		
Race	808				
White	726				
Black or African American	82	1.004 (0.711-1.419)	0.981		

### Analysis of the Prognosis Value of MiRNA-30a-5p in LUAD

**Figure 6:**
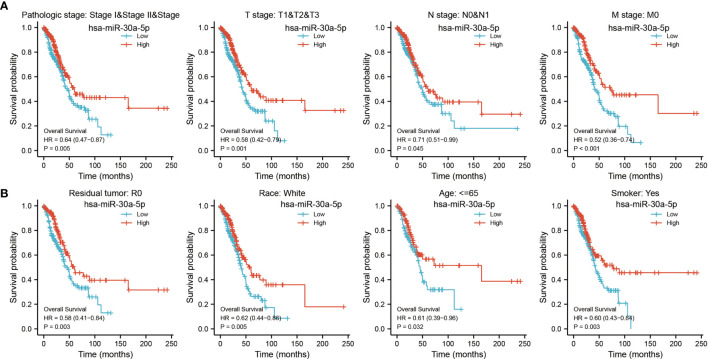
Prognostic value of differential expression of miRNA-30a-5p in different subgroups. Prognostic value of miRNA-30a-5p in diverse subgroups, including **(A)** pathologic stage and TNM stage, **(B)** smoking, residual tumors, age, and ace. CR, complete response; PR, partial response; SD, stable disease; PD, progressive disease.

**Figure 7:**
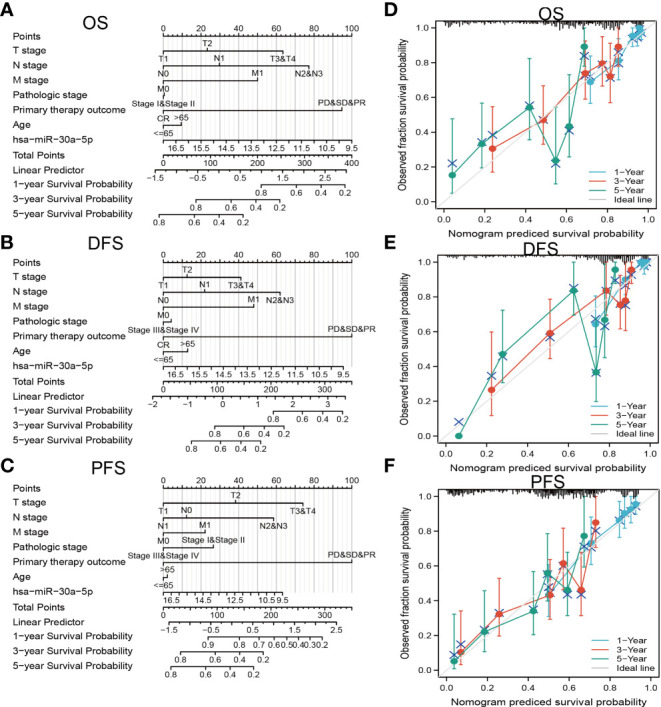
Construction and performance validation of the miRNA-30a-5p based nomogram for LUAD patients. Nomogram to predict overall survival (OS) **(A)**, disease-free survival (DFS) **(B)**, and progression-free survival (PFS) **(C)** for lung cancer patients. The calibration curve and Hosmer–Lemeshow test of nomograms in the TCGA-LUAD cohort for **(D)** OS, **(E)** DSS, and **(F)** PFI.

We examined the prognostic value of miRNA-30a-5p in LUAD patients based on different subgroups, mainly comprise of the different pathological stage, TNM stage, smoker, residual tumor, age and race groups. Results confirmed that decreased miRNA-30a-5p expression was correlated with adverse clinical outcomes in diverse groups (**Figures 6A, B**). To accurately predict the 1-, 3-, and 5-year overall survival (OS), disease-free survival (DFS), and progression-free survival (PFS) in LUAD patients, we used miRNA-30a-5p expression and (TNM stage, age, and pathologic stage) to construct a nomogram. Result confirmed that this nomogram could predict the overall survival (OS), disease-free survival (DFS), and progression-free survival (PFS) of LAUD patients (**Figures 7A–F**). To sum up, this nomogram may be a model for predicting the overall survival (OS), disease-free survival (DFS), and progression-free survival (PFS) in LUAD patients with miRNA-30a-5p.

### Overexpression of MiRNA-30a-5p Inhibits Proliferation and Migration of LUAD Cells

**Figure 8:**
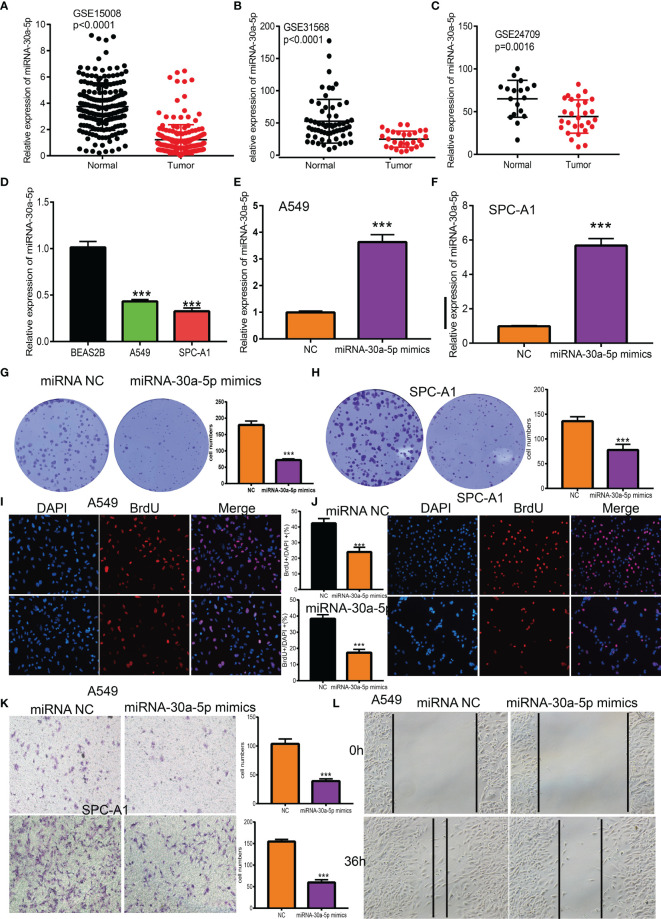
Over-expression of miRNA-30a-5p inhibits the cell proliferation and migration ability of LUAD cells. **(A–C)**The expression of miRNA-30a-5p in LUAD was examined by GEO datasets. **(D)** The expression of miRNA-30a-5p in LUAD cell lines was examined by qRT-PCR assay. **(E, F)** The expression of miRNA-30a-5p in LUAD cells lines after over-expression of miRNA-30a-5p was examined by using the qRT-PCR assay. **(G–J)** Over-expression of miRNA-30a-5p on cell growth ability examined by clone information and BrdU assays. **(K, L)** Over-expression of miRNA-30a-5p on cell migration ability examined by transwell and wound healing assay. Quantification data were also indicated. Scale bar=50 μm. ***P < 0.001.

To determine the expression of miRNA-30a-5p in lung cancer, we examined the GEO dataset found that miRNA-30a-5p was decreased in lung cancer tissue and peripheral blood serum (**Figures 8A–C**). Next, we utilized the qRT-PCR assay to examine the expression of miRNA-30a-5p in diverse LUAD cells line, these data imply that miRNA-30a-5p was down-regulated in LUAD cells than the control normal lung cells (**Figure 8D**). Given the low expression of miRNA-30a-5p in LUAD tissues, we speculate that miRNA-30a-5p might play suppresses role in the pathogenesis of LUAD. To investigate the functional roles of miRNA-30a-5p in LUAD cells, we transiently overexpressed miRNA-30a-5p mimics in A549 cell, the expression of miRNA-30a-5p after overexpression was confirmed by qRT-PCR assay. As expected, the result suggested that increasing the expression of miRNA-30a-5p after over-expression of miRNA-30a-5p (**Figures 8E, F**). Next, we performed the function assay to examine the over-expression of miRNA-30a-5p on the cell proliferation and migration ability of LUAD cells. The result indicated that elevated miRNA-30a-5p expression was significantly inhibited the cell proliferation and migration abilities of LUAD cells (**Figures 8G–L**). Collectively, these data imply that miRNA-30a-5p plays tumor suppressor role in the LUAD progression.

### MiR-30a-5p Inhibits Cancer Stem Cell Maintenance in LUAD

**Figure 9:**
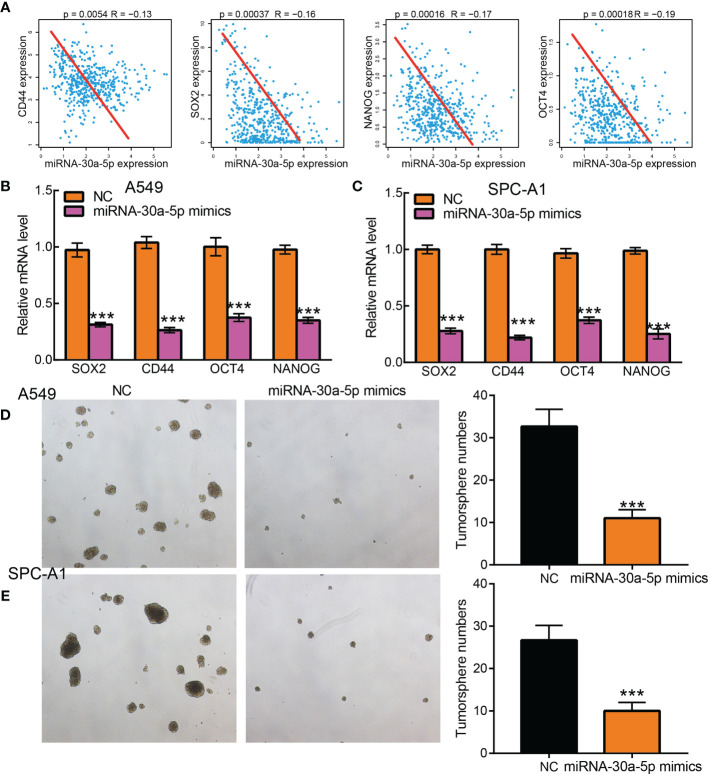
Over-expression of miRNA-30a-5p inhibits cancer stem cell maintenance in LUAD. **(A)** The correlation between miRNA-30a-5p and cancer stem cell maintenance-related factors, including SOX2, CD44, OCT4, and NANO in TCGA-LUAD, was examined using Pearson’s correlation analysis. **(B, C)** Relative mRNA expressions of cancer stem cell marker genes, including CD44, OCT4, SOX2, NANOG in A549, and SPC-A1cells, were examined by Real-time RT-PCR upon over-expression of miRNA-30a-5p. **(D, E)** Tumor sphere formation abilities of indicated cells after over-expression of miRNA-30a-5p were examined by tumorsphere assay. Scale bar=50 μm. . ***P < 0.001.

Previous KEGG analysis show that miR-30a-5p target gene may participate in the Wnt signaling pathway, to further characterize the function of miR-30a-5p regulates LUAD cancer stem cell maintenance, we performed correlation analysis and showed that miR-30a-5p negative correlated with the expressions of well define cancer stem cell marker genes, including CD44, Sox2, Oct4 and NANOG (**Figure 9A**) (14). We showed that forced miR-30a-5p expression was reduced the expression of stem cell marker genes in LUAD cells (**Figures 9B, C**). Furthermore, we revealed that over-expression of miR-30a-5p inhibits the lung cancer stem cell self-renewal ability (**Figures 9D, E**). Above all, these results demonstrated that miR-30a-5p plays an important role in regulating LUAD cancer stem cell maintenance.

### Correlation Analysis Between MiRNA-30a-5p Expression and Infiltrating Immune Cells

**Figure 10:**
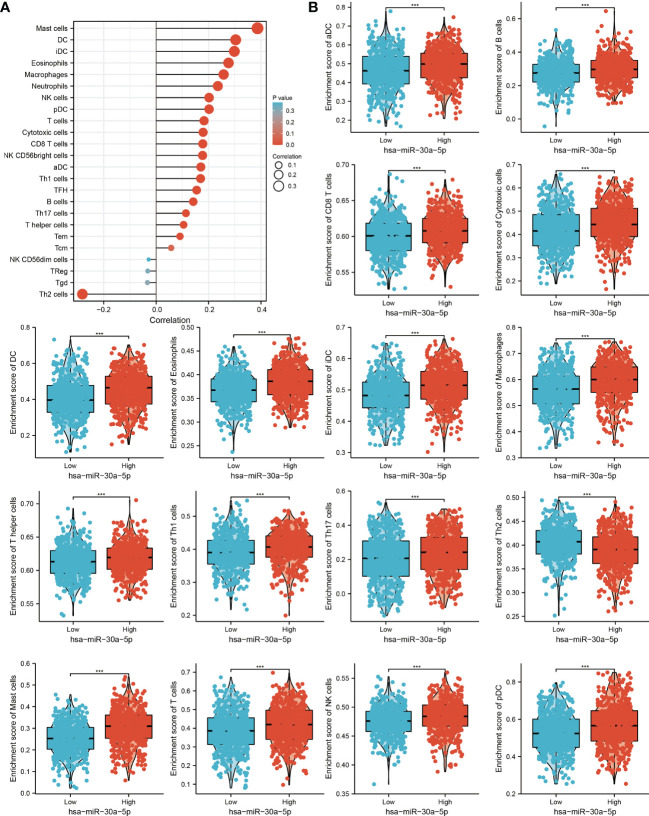
Analysis of the correlation between miRNA-30a-5p expression and immune infiltration. **(A)** Correlation between the relative abundances of 24 immune cells and miRNA-30a-5p expression level. **(B)** Diverse proportions of immune cell subtype in tumor samples in high and low miRNA-30a-5p expression groups.

**Figure 11:**
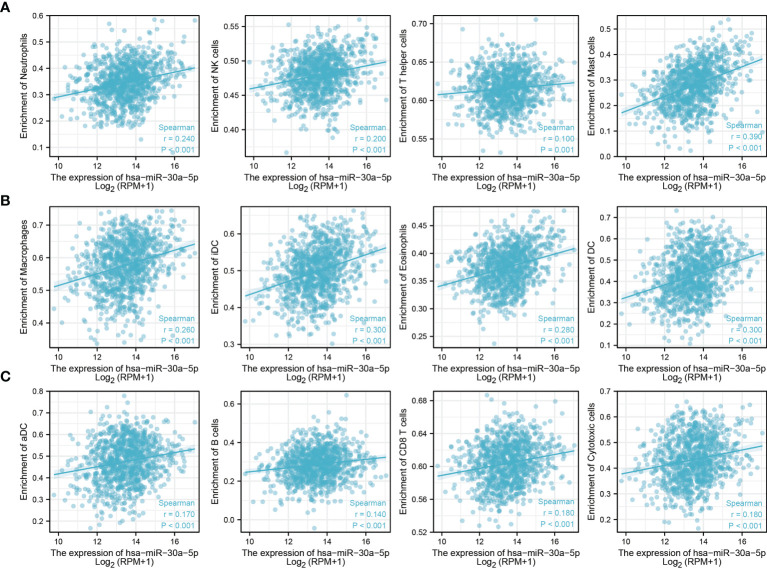
Analysis the correlation between miRNA-30a-5p expression and diverse immune infiltration. **(A–C)** Correlation between miRNA-30a-5p and various immune infiltrate in LUAD.

Considering miRNA-30a-5p plays crucial roles in the immune response and progression of lung cancer. We explored the correlation between the expression of miRNA-30a-5p and immune infiltration in LUAD by using Spearman correlation, the analysis data demonstrated that miRNA-30a-5p positively correlated with the immune infiltration of mast cells, DC, IDC, eosinophils, macrophages, neutrophils, NK cells, pDC, T cells, cytotoxic cells, CD8 T cells, NK, CD56bright cells, ADC, Th1 cells, TFH, B cells, Th17 cells, T helper cells and Tem in LUAD (**Figures 10** and **11**). These results demonstrated that miRNA-30a-5p plays a significant role in the immune response of LUAD.

### Kaplan-Meier Survival Curves According to High and Low Expression of MiRNA-30a-5p in Immune Cell Subgroups in LUAD

**Figure 12:**
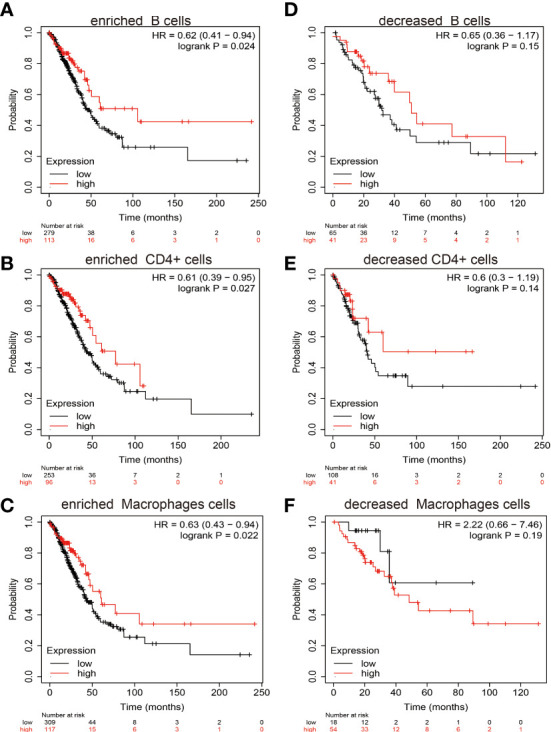
Overall survival curves based on the expression of miRNA-30a-5p in the immune cell subgroups in LUAD. **(A–F)** Correlation between miRNA-30a-5p expression and overall survival in different immune cell infiltration groups in LUAD patients.

Since miRNA-30a-5p expression was significantly positive with diverse immune cells infiltration. We next studied the expression of miRNA-30a-5p and diverse immune cells infiltrate whether affecting the prognosis of LUAD patients. We uncover that increased miRNA-30a-5p expression and enriched the B cells, CD4+ cells and Macrophages cells will indicate a better prognosis. While high expression miRNA-30a-5p and decreased the B cells, CD4+ cells, and macrophages were correlated with poor clinical outcomes (**Figure 12**).

## Discussion

Lung adenocarcinoma is a malignant tumor characterized by uncontrolled growth of cells in the lung and bronchus (15). The clinical LUAD outcomes are far from satisfactory using current treatments. Therefore, it is crucial to find stable potential biomarkers to predict prognosis and guide individualized therapies (12). Accumulating evidence has indicated that miRNAs play indispensable roles in cancer progression and drug resistance, while the potential molecular mechanism of miRNA-30a-5p in the tumor microenvironment (TME) is still unclear. MiRNAs *via* regulation the expression of oncogenes or tumor suppressors and participate in diverse cancer development. For instance, it has been shown that elevated the expression of microRNA-30a-5p could reduce liver cancer cell growth and promotes cell apoptosis *via* inhibiting the MTDH/PTEN/AKT signaling pathway (16). Recently, a new opinion has emerged that miR-30a-5p inhibits tumor growth *via* down-regulation of the expression of denticles protein homolog in colon carcinoma (17). In renal cancer, the study has been reported that MicroRNA−30a−5p inhibits the MTDH/PTEN/AKT pathway and leads to reducing the tumor cell proliferation of human renal cancer (18). In this study, we analyzed miR-30a-5p expression, prognostic value, genetic variations, and correlation with tumor immune cell infiltration in LUAD for the first time.

In the present study, we found that miRNA-30a-5p significantly decreased in BLCA, THCA, STAD, PRAD, LUSC, LUAD, KIRP, KICH, HNSC, ESCA, and BRCA, and low expression of miRNA-30a-5p was associated with the poor prognosis of diverse cancer. More importantly, we found that miRNA-30a-5p expression was significantly decreased in LUAD cells lines, LUAD cancerous tissues, and blood serum, miRNA-30a-5p lower expression patients exhibit worse clinical outcome compared to the patients with higher miRNA-30a-5p expression, decreased expression of miRNA-30a-5p was associated with advanced clinical pathologic characteristics, including the TNM stage, pathologic stage, Gender, Age Primary therapy outcome, and smoking. ROC curve analysis of miRNA-30a-5p showed an AUC value of 0.902, indicating its prognostic value in LUAD. These results suggest that miRNA-30a-5p plays an important role in the progression and metastasis of LUAD. Our findings are consistent with previous researches. MiRNA-30a-5p was down-regulated in some tumor tissues and associated with clinic-pathological features, including late T stage, lymph nodal metastasis, and TNM staging (18, 19). Our results strongly indicate that miRNA-30a-5p can be used as a prognostic biomarker for LUAD. Thus, our study provides new insights into understanding the potential roles of miRNA-30a-5p in LUAD progression and its potential use as cancer prognostic biomarker.

Mechanically, GSEA and KEGG enrichment analysis revealed that miRNA-30a-5p target genes expression was largely enriched in various cell proliferation and immune response-related pathways, such as non-small cell lung cancer, apoptosis, chemokine signaling pathway, cytokine-cytokine receptor interaction, ECM receptor interaction, INFγ mediated phagocytosis, JAK-STAT signaling pathway, MAPK signaling pathway, Natural killer cell-mediated cytotoxicity, NOD-like receptor signaling pathway, T cell receptor signaling pathway, and Toll-like receptor signaling pathway. Above all, these results demonstrated that miRNA-30a-5p expression might be plays an important role in cancer progression and immune response regulation. Therefore, targeting miRNA-30a-5p seems to be an alternative strategy for tumor therapy.

Immunotherapy to boost T cell functionality in tumors is rapidly becoming standard treatment (20). In lung cancer, tumor-infiltrating CD4+ T cells play an essential role in the immune response (21). Here, we first showed that high miRNA-30a-5p expression in LUAD is associated with the increased infiltration of mast cells, DC, IDC, eosinophils, macrophages, neutrophils, NK cells, pDC, T cells, cytotoxic cells, CD8 T cells, NK, CD56bright cells, ADC, Th1 cells, TFH, B cells, Th17 cells, T helper cells, and Tem. These results may explain that high expression of miRNA-30a-5p partly affects the prognosis of LUAD patients through immune infiltration. Therefore, our result demonstrated that miRNA-30a-5p might affect immune cell infiltration and immunotherapy efficacy, which makes them a predictive biomarker for immunotherapy in LUAD patients.

More importantly, miRNA-30a-5p associated with the prognosis of LUAD patients partially through immune cell infiltration. These findings indicate that miRNA-30a-5p could be a novel immune-related therapeutic target in LUAD. Finally, we identified that miRNA-30a-5p can suppress cell proliferation, colony formation, migration, and self-renewal capability in LUAD cells. According to our results, miRNA-30a-5p may be useful as a serum biomarker for the early detection of LUAD patients.

This study improves our understanding of the correlation between miRNA-30a-5p and LUAD, but some limitations still exist. First, although we explored the correlation between miRNA-30a-5p and immune infiltration in LUAD patients, there is a lack of experiments to validation the function of miRNA-30a-5p in the tumor microenvironment regulation of LUAD. Second, we uncover that depletion of miRNA-30a-5p was inhibits cell proliferation and cell migration of LUAD cells. However, the molecular mechanisms of miRNA-30a-5p in tumor growth and metastasis need to be explored in further studies. Third, we did not conduct the *in vivo* experiments to validation the function of miRNA-30a-5p in the tumor metastasis and tumor microenvironment regulation of LUAD. In the future, we will further study the function of miRNA-30a-5p in tumor metastasis and tumor microenvironment regulation of LUAD.

## Conclusions

In summary, we found that miRNA-30a-5p expression was significantly positive with immune cell infiltration. Moreover, miRNA-30a-5p acts as a tumor suppressor gene in LUAD cell growth and migration. We also revealed that miRNA-30a-5p expression can serve as a useful biomarker for lung cancer prognosis.

## Data Availability Statement

The original contributions presented in the study are included in the article/supplementary material. Further inquiries can be directed to the corresponding authors.

## Author Contributions

XJ and YY designed this work, performed experiments. LT, JW, and DZ analyzed data. WC and LD write and revised the manuscript. All authors have read and approved the final version of the manuscript.

## Funding

This work was supported by the National Nature Science Foundation of China (82160508), Yunnan Applied Basic Research Projects (YNWRMY-2019-067), Yunnan Province Specialized Training Grant for High-Level Healthcare Professionals (D-201614), and Yunnan Province Applied Basic Research Foundation (2019FE001) to LD.

## Conflict of Interest

The authors declare that the research was conducted in the absence of any commercial or financial relationships that could be construed as a potential conflict of interest.

## Publisher’s Note

All claims expressed in this article are solely those of the authors and do not necessarily represent those of their affiliated organizations, or those of the publisher, the editors and the reviewers. Any product that may be evaluated in this article, or claim that may be made by its manufacturer, is not guaranteed or endorsed by the publisher.
