# Corrigendum: Identification and Validation Prognostic Impact of miRNA-30a-5p in Lung Adenocarcinoma

**DOI:** 10.3389/fonc.2022.862076

**Published:** 2022-02-25

**Authors:** Xiulin Jiang, Yixiao Yuan, Lin Tang, Juan Wang, Dahang Zhang, William C. Cho, Lincan Duan

**Affiliations:** ^1^The Department of Thoracic Surgery, The Third Affiliated Hospital of Kunming Medical University, Kunming, China; ^2^Key Laboratory of Animal Models and Human Disease Mechanisms of Chinese Academy of Sciences, Kunming Institute of Zoology, Kunming, China; ^3^Department of Clinical Oncology, Queen Elizabeth Hospital, Hong Kong, Hong Kong SAR, China

**Keywords:** miRNA-30a-5p, LUAD, prognosis biomarker, cell proliferation, cell migration

**Figure 5I**. miRNA-30a-5p was down-regulated in the sera of LUAD using qRT-PCR assay, at which it is a reverse of the two groups in the previous **Figure 5I**. **Figure 8B**, it seems that the labels of the X-axis being masked in the previous **Figure 8B**. The corrected **Figure 5** and **Figure 8** are shown as below.

**Figure 5:**
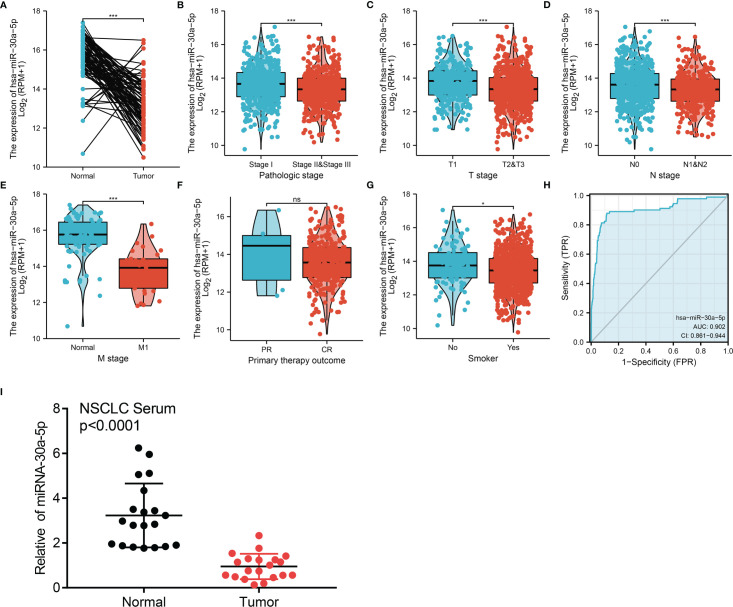
miRNA-30a-5p was down-regulated in LUAD. **(A)** MiRNA-30a-5p was down-regulated in lung cancer based on TCGA-LAUD dataset. **(B–G)** Correlation between miRNA-30a-5p and clinicpathologic features, including the pathology stage, TNM stage, smoker, and therapy outcome in LUAD. **(H)** ROC curve analysis of miRNA-30a-5p showed an AUC value of 0.902 in LUAD. **(I)** miRNA-30a-5p was down-regulated in peripheral blood serum of LUAD analysed using qRT-PCR assay. *P < 0.05, ***P < 0.001. CR, complete response; PR, partial response; SD, stable disease; PD, progressive disease. NS: p >0.05.

**Figure 8:**
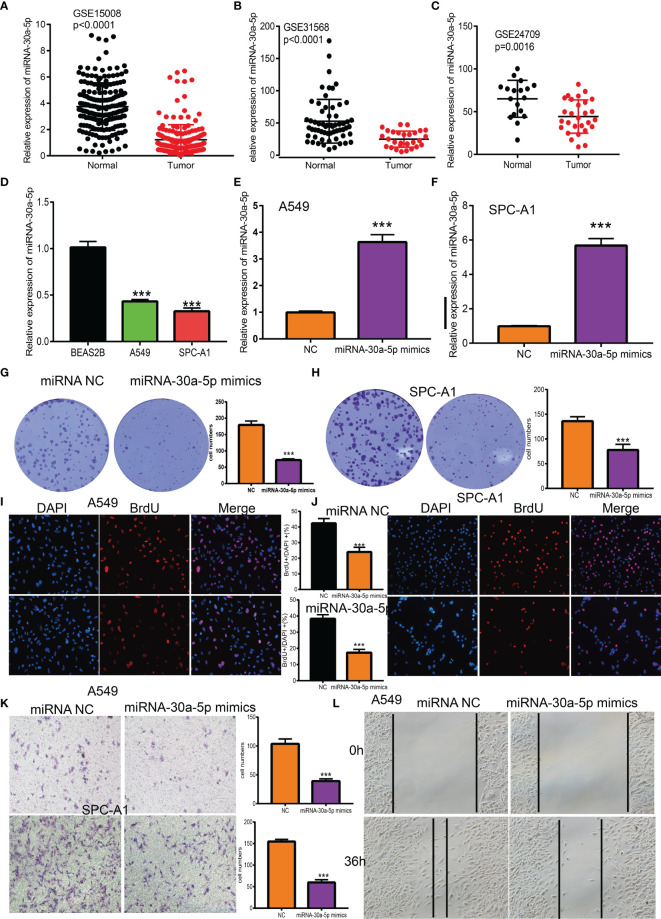
Over-expression of miRNA-30a-5p inhibits the cell proliferation and migration ability of LUAD cells. **(A–C)** The expression of miRNA-30a-5p in LUAD was examined by GEO datasets. **(D)** The expression of miRNA-30a-5p in LUAD cell lines was examined by qRT-PCR assay. **(E, F)** The expression of miRNA-30a-5p in LUAD cells lines after over-expression of miRNA-30a-5p was examined by using the qRT-PCR assay. **(G–J)** Over-expression of miRNA-30a-5p on cell growth ability examined by clone information and BrdU assays. **(K, L)** Over-expression of miRNA-30a-5p on cell migration ability examined by transwell and wound healing assay. Quantification data were also indicated. Scale bar=50 mm. ***P < 0.001.

The authors apologize for these errors and state that this does not change the original scientific conclusions of the article. The original article has been updated.

## Publisher’s Note

All claims expressed in this article are solely those of the authors and do not necessarily represent those of their affiliated organizations, or those of the publisher, the editors and the reviewers. Any product that may be evaluated in this article, or claim that may be made by its manufacturer, is not guaranteed or endorsed by the publisher.

